# Lived experiences of heat stress among migrant agricultural workers in Spain: a qualitative study

**DOI:** 10.1186/s12939-026-02850-x

**Published:** 2026-04-11

**Authors:** Lena van Selm, Caroline Voorhis, Sarah Williams, Daniel Izuzquiza-Regalado, Sebastian Notario-Gallego, Seydou Diop, Stella Evangelidou, Erica Briones-Vozmediano, Ana Requena-Méndez

**Affiliations:** 1 Barcelona Institute for Global Health (ISGlobal), Barcelona, Spain; 2 Faculty of Medicine and Health Sciences, University of Barcelona, Barcelona, Spain; 3 Servicio Jesuita a Migrantes (SJM), Almería, Spain; 4 Pagesos Solidaris, Unió de Pagesos, Lleida, Spain; 5 Asociación Nueva Ciudadanía por la Interculturalidad (ASNUCI), Lepe, Spain; 6 Department and Faculty of Nursing and Physiotherapy. Consolidated Research Group in Society, Health, Education and Culture (GESEC), University of Lleida, Lleida, Spain; 7 Research Group in Healthcare (GRECS), Lleida Biomedical Research Institute, Dr. Pifarré Foundation (IRBLleida), Lleida, Spain; 8 Department of Medicine Solna, Karolinska Institutet, Stockholm, Sweden; 9 CIBERINFEC, ISCIII - CIBER de Enfermedades Infecciosas, Instituto de Salud Carlos III, Centro de Investigación Biomédica en Red de Enfermedades Infecciosas, Madrid, Spain

**Keywords:** Heat stress disorders, Occupational exposure, Occupational health, Outdoor workers, Transients and migrants

## Abstract

**Background:**

Agricultural workers face high heat stress risk due to environmental and working conditions. Migrant agricultural workers (MAW) are especially vulnerable due to additional structural factors including irregular migratory status and high economic needs. Our study explores the lived experiences of occupational heat stress among MAW and its impact on health.

**Methods:**

An interpretive, qualitative design was employed. Through purposive sampling we selected MAW in Almeria, Lleida, and Huelva for semi-structured interviews. Interviews were audio-recorded, transcribed, and analyzed using reflexive thematic analysis.

**Results:**

Thirty interviews were conducted with six female and 24 male MAW, predominantly from sub-Saharan and North-Africa. The findings were organized into five overarching themes: Working under the sun: exposure and embodiment; The dilemma of heat: work or wellbeing; Employer´s efforts to protect workers: a regime of discretion; Protecting oneself from the heat: navigating limited agency; and non-work-related factors: compounding vulnerabilities. Participants described discomfort and heat-related illness symptoms. Protective strategies included drinking water, wearing appropriate clothing, and taking breaks, but pressure from supervisors to maintain a fast work pace and limit breaks often compromised these protective efforts. Employer discretion largely determined access to heat-protective measures. Participants also reported limited ability to cool down after work due to high indoor temperatures in substandard housing.

**Conclusions:**

Occupational heat risk for MAW extends beyond environmental factors and is amplified by socioeconomic inequities. Implementation of more specific and enforceable heat-protection measures and improved enforcement mechanisms, alongside mandated improvements to working and living conditions, are needed to reduce heat-related health risks.

## Background

Climate change is amplifying heat exposure, resulting in more frequent, longer-lasting, and more intense heat waves, thereby posing substantial threats to human health and ecosystems [1]. Agricultural and other outdoor workers are particularly vulnerable to heat stress due to combined environmental exposures such as high temperatures, direct sunlight, and humidity [2]. This risk is further exacerbated by the physically demanding nature of many tasks, which increase metabolic heat generation [3]. Occupational heat stress can manifest in heat-related illness (HRI) symptoms, ranging from less severe ones, such as dizziness or muscle cramps, to severe symptoms such as fainting [4, 5]. Moreover, it increases the risk of occupational injuries and can lead to death due to worsening of pre-existing health conditions [2, 3, 6–9]. In the long term, heat stress can cause acute kidney disease and can progressively impair kidney function, contributing to chronic kidney disease [6, 8, 10]. 

Agriculture is among the sectors with the highest risk of heat stress, given its combination of physical exertion and outdoor exposure [2, 11]. In the United States, farmworkers are nearly 20 times more likely to die from heat exposure than other workers [12]. In Spain, agriculture had the strongest association between increase in daily mean temperature and work-related injuries [13], while agricultural workers in Italy had a 13% higher injury risk on hot days [14]. 

Migrants are disproportionately represented in agricultural jobs. In 2019, Spain had the highest proportion of migrants employed in agriculture within the EU [15]. Accurate numbers on MAW in Spain are scarce. In 2024, 35% (233,114 people) of workers affiliated with Spain´s Special Agricultural System (Sistema Especial Agrario; SEA) were foreign nationals, compared with 13.4% of migrants living in Spain [16–18]. These numbers are an underestimation, as the SEA does not include workers who worked less than 30 days and does not always capture mobility between regions [19]. When looking at 2024 data from the State Public Employment Service (Servicio Público de Empleo Estatal; SEPE), 50.7% of all contracts were issued to foreign nationals, a proportion that was considerably higher in specific provinces, including Lleida (88%), Almeria (77%) and Huelva (75%) [20]. Finally, none of these figures include undocumented migrants working without a formal contract. In 2019, the International Labor Organization estimated that 37.8% of Spain’s agricultural workforce was informal [21]. Using the SEA data as a starting point, this would add over 400,000 people to the agricultural workforce, the majority of whom are expected to be (undocumented) migrants, meaning that the estimated proportion of migrants working in agriculture in Spain could reach up to 60%.

Multiple factors heighten migrant agricultural workers’ (MAW) vulnerability to HRI. For example, factors such as low education levels, irregular migratory status and high economic needs can force migrants to accept low-paid, high-risk jobs [22]. In addition, fear of losing work can make MAW take more risks, including taking less breaks and working faster despite occupational heat exposure [23]. A study from Cyprus showed that MAW had worked faster, took fewer breaks and had higher levels of occupational heat stain than native workers [24]. Limited language proficiency, cultural barriers, and minimal control over workplace conditions further increase injury and illness risk [5, 25]. In Spain, many MAW live in precarious situations, including overpopulated housing in remote areas [26, 27]. In Andalusia, many MAW, especially when undocumented, live in shanty towns close to the agricultural sites, often without access to running water and electricity [28–30]. Finally, MAW, especially with an irregular status, frequently move between regions for seasonal employment, facing persistent instability [31]. 

While most research on occupational heat among MAW originates from the United States [23], evidence from Europe remains scarce, even though the Mediterranean is recognized as a climate change hotspot, with temperatures rising faster than the global average [32]. In Spain, only two studies have directly measured occupational heat stress among farmworkers: one in Alicante, where 21% of male workers were exposed at a mean wet bulb globe temperature (WBGT) of 24.5 °C [33], and another in Huelva, where 26% of female workers experienced heat strain at a mean WBGT of 25.2 °C [34]. 

To date, no previous studies have focussed on the experience of MAW with heat in Spain. Our study therefore aims to explore the lived experiences of occupational heat stress among MAW in Spain. By voicing their narratives, this research intends to contribute to broader discussions on labor rights, social justice, and the development of fair and inclusive heat adaptation policies in the agricultural sector.

## Methods

### Study design

This study used a participatory, qualitative design with an interpretive approach. The participatory part consisted of the creation of a core stakeholder group, including one civil society stakeholder from each study site, to provide input throughout the study. All three core stakeholder group members were part of associations working with MAW and one was a former MAW. Throughout the participatory recruitment process we closely collaborated with the core stakeholder group to ensure access to MAW study participants.

### Study sites

Data were collected in the provinces of Almeria, Huelva and Lleida, which are three of the main agricultural sites in Spain, each having seasons with high outdoor temperatures. Data collection took place in July 2024 in Almeria (3 municipalities), August 2024 in Lleida (8 municipalities) and May 2025 in Huelva (5 municipalities). Almeria is Europe´s main hub of intensive agriculture in greenhouses and the main crops that are being cultivated are tomatoes, bell peppers, zucchini, cucumber [35, 36]. Huelva is the main berry producer of Europe and most of the work is carried out in greenhouses [37]. Lleida is Catalonia’s biggest agricultural sites and the cultivation of crops, such as stone fruits and cereals, is carried out outdoors [38]. Spanish social security data show the number of foreign workers affiliated with the Special Agricultural System (*Sistema Especial Agrario*) [39]. Between January and September 2025, the monthly averages were 36,500 foreign workers in Almeria, 20,500 in Huelva and 11,100 in Lleida [39]. However, these numbers do not include informal workers, which in Almeria only were estimated to be 25,000 [40]. In Almeria and Huelva an estimated 7000 and 5000 people, respectively, are living in informal settlements, mainly consisting of undocumented workers [41]. Working conditions vary according to the type of employment contract. Three main categories can be distinguished: (1) workers formally contracted within Spain, either on a fixed or seasonal basis; (2) workers recruited abroad under temporary migration schemes that allow them to work in Spain for up to nine months during the agricultural season; and (3) workers without formal contracts, who are employed informally [42]. The second contract type is widely used in Huelva and Lleida, as the work is seasonal, but not in Almeria, where the work continues all year round.

### Participant recruitment

This study focused exclusively on MAW, considering that migrants constitute a substantial proportion of the agricultural workforce and might face migrant-specific HRI risk factors. A purposive sampling strategy was used in which eligible participants were identified with the support of local civil society organizations, some of them migrant-led, working with migrant agricultural workers who facilitated the contact in many cases. In Huelva a female Moroccan translator, from a collaborating NGO, supported with the recruitment of Moroccan women. Criteria for participation were: being foreign-born, working in agriculture in one of the three study sites, being over 18 years old, speaking sufficient Spanish or English to engage in a meaningful conversation, and provided written consent to participate. While selecting participants we aimed for a balanced sample intended to represent the target population in terms of gender and country of origin.

### Data collection procedure

A draft topic guide was developed based on the scientific literature. Subsequently, an online participatory workshop was conducted with the core stakeholder group members and additional relevant stakeholders from the study sites, representing or working closely with MAW; several of these stakeholders were proposed by the core group members. Workshop participants included five NGO employees working with MAW, one migrant agricultural worker, and one external researcher. The workshop aimed to gather input on the research topics to ensure their relevance to the local contexts. Based on this input, the final interview guide was developed, which included questions about lived experiences of occupational heat, the effects of heat on work, wellbeing and health, and access to healthcare services (see Annex 1).

At the beginning of each interview, the study was explained and the participants signed an informed consent form and a voice rights transfer form. Participants received a €20 voucher for a local supermarket after participation. Interviews were conducted by LvS in Spanish or English and were audio recorded, with durations ranging from 22 to 56 min. As these were mostly not the native languages of the participants, the interviewer regularly sought confirmation from participants and, when necessary, reformulated questions to ensure accurate understanding. Two interviews with Moroccan women were conducted in Darrisha with the help of a translator from a local NGO. Interviews took place at the homes of the participants, spaces provided by the civil society organizations or when no other space was available outdoors in a place as private as possible. Data collection continued until data saturation was reached, meaning that no new insights were emerging from additional interviews. During data collection and analysis, participant anonymity was ensured.

### Data analysis

Audio files were transcribed using Happy Scribe Pro for, a GDPR-compliant transcription service that provides secure processing of audio data. The initial machine-generated transcripts were then checked and corrected by a member of the research team for accuracy. Transcripts were analyzed in the language in which the interview was conducted (Spanish, English or Darrisha with translation to Spanish). The responses in Darrisha were translated to English by a member of the research team, to ensure the quality of the translations by the translator. Transcripts were uploaded into Atlas.ti, and coding was carried out by two researchers. The analytic process was informed by Braun and Clarke’s reflexive thematic analysis [43, 44], and involved repeated reading for familiarization, systematic coding, and iterative theme development. An inductive coding strategy was applied, in which data was organized in sub-topics based on the interview guide and at the same time codes were developed that identified patterned meanings across the dataset. After initial coding, descriptive summaries were written for each code to capture key features of the data. These texts provided a foundation for comparing and reorganizing data, which facilitated the development of broader themes representing shared meanings. The process was iterative rather than strictly linear, involving movement back and forth between coding, writing, and theme refinement.

Analysis was led by LvS, who iteratively developed draft themes, that were discussed and finetuned within the research team to enhance reflexivity. In line with a reflexive approach, themes are understood as the product of the researcher’s active engagement with the data rather than entities to be ‘discovered’ [44]. The participants’ quotes included in this paper were translated by LvS and revised by the co-authors; Annex 2 shows the original quotes in Spanish. The study was conducted and reported using the Consolidated Criteria for Reporting Qualitative Research (COREQ; Appendix 1) [45]. 

### Researcher reflexivity

LvS who conducted the interviews, has a background in public health, with prior experience in qualitative research and in working with migrant populations in Spain. As a non-migrant researcher, she approached the study as an outsider, which required careful attention to building trust and rapport with participants. Discussions with the civil society organization members, during the data collection phase, and with co-authors during the data analysis phase, helped to critically examine interpretations and ensure that interpretations and emerging themes reflected participants’ perspectives rather than the researcher’s preconceptions. This reflexive approach aimed to enhance the credibility and transparency of the study.

### Ethical approval

#### Etlhical approva

was obtained from the Hospital Clinic ethical committee with code HCB/2024/0015.

## Results

A total of 30 individual semi-structured interviews were conducted (Table 1). Participants were born in Morocco (8), Senegal (8), Colombia (4), Gambia (2), Mali (2), Democratic Republic of Congo (1), Guinea-Bissau (1), Pakistan (1) and Romania (1). Most were male (80%) and ages ranged from 18 to 58 years. Most participants [17] had a residence and working permit in Spain, eight participants did not have a residence or working permit, six workers were contracted in their country of origin to perform seasonal work in Spain, and one was an asylum seeker working while awaiting the resolution.

**Table 1:** Participant characteristics

	Almeria (*n* = 12)	Huelva (*n* = 8)	Lleida (*n* = 10)
**Area of origin**			
North Africa	4	4	-
Sub-Saharan Africa	8	4	4
South America	-	-	4
Other	-	-	2
**Gender**			
Male	12	4	8
Female	-	4	2
**Age group** ^**a**^			
< 30 years	9	1	2
30–40 years	3	2	3
> 40 years	-	1	3
**Working permit**			
Yes	6	4	9
No	6	2	1
Seasonal^b^	-	2	
**Housing**			
Apartment	9	4	1
Temporary housing for the season^c^	-	6	3
Substandard housing / homeless	3	-	4

a: For six participants the age was missing; b: Allowed to work in Spain for a limited amount of time each year before returning to country of origin; c Farmhouses provided by the employer or municipality hostel

During the reflexive thematic analysis, five themes and eleven sub-themes were identified (Table 2).

**Table 2:** themes and sub-themes

Theme	Sub-theme
1. Working under the sun: Exposure and embodiment	Familiar but harsh: normalizing extreme conditions
	Embodied consequences of heat
	Unequal vulnerability to heat-related effects
2. The dilemma of heat: Work or wellbeing	Adverse incentives and economic precarity
	Safeguarding wellbeing: Prioritizing health over work
3. Employer´s efforts to protect workers: A regime of discretion	Contingent protection
	The “Good Boss” / “Bad Boss” dichotomy
4. Protecting oneself from the heat: Navigating limited agency	
5. Non-work-related factors: Compounding vulnerabilities	Heat at home: Precarious housing conditions
	The intersection of legal status and heat-risk

### Theme 1: Working under the sun: perceptions and health impacts

#### Familiar but harsh: normalizing extreme conditions

Most participants described Spanish summers as extremely hot but considered themselves accustomed to high temperatures. Except for one Colombian worker from a cooler climate, all participants reported coming from countries with high heat exposure.

*Well*,* I can stand it because I’m African. We already have heat in Africa all year round. It’s summer all year. So the heat doesn’t surprise me*,* it doesn’t surprise me*.

- Male, Lleida 4

Despite this sense of habituation, participants across all sites acknowledged that summer heat made agricultural work difficult. In Almeria and Huelva, many participants emphasized that plastic greenhouse coverings intensified temperatures.

*Because the plastic makes it very hot. If it’s 30 degrees outside*,* inside it’s almost 40*

- Male, Huelva 1

Workers in Almeria noted that old-design greenhouses with low roofs get particularly hot. Perceptions varied by site: in Almeria, participants widely agreed that working in the heat was very hard; in Huelva, some workers from Mali felt more accustomed to it, while others described severe discomfort.

*You feel terrible. You wait for your shift to end and you come running home to shower and rest. That’s it. All day we pour water over our heads*,* go in and out like that. Everything suffers from the heat*,* but what can we do? We have to work*.

- Female, Huelva 5

In Lleida, heat was often viewed as an inevitable aspect of the job rather than a persistent problem, though some recalled extreme days when conditions became intolerable.

*Last year it was bad*,* pretty bad. A lot of heat stroke*,* a lot of heat. And well*,* it came out in the news that*,* even deaths*,* there was one in the news. And one day we were working*,* that day*,* I mean*,* I had to drink water and look for shade because I couldn’t take it anymore*.

- Male, Lleida 1

#### Embodied consequences of heat

Workers described a range of physical symptoms during hot periods, most commonly headaches, dizziness, and fatigue. In Lleida and Huelva these symptoms occurred sporadically, while in Almeria they were reported more frequently. Most workers said symptoms subsided after cooling down, though a few described persistent effects throughout the day. One participant explained that his headache would continue after work due to the heat at home.

*Well*,* during hot times you can sometimes work in the field*,* it’s very hot*,* your head hurts*,* well*,* arriving home you also find heat*,* so your head can keep hurting*,* and then you can take pills so you can sleep a little*.

- Male, Lleida 1

Several workers mentioned feeling close to fainting, though none reported actually fainting. Others described heavy sweating, dehydration, accelerated heartbeat, and memory lapses:

*Inside the greenhouse it’s like a shock*,* like working without… Sometimes when working you don’t know what you’re doing*,* you just work*,* because it’s so hot*.

- Male, Almeria 12

Some participants reported being sick or feverish for days after working on an extremely hot day. Others mentioned blurred vision, joint pain, or breathing difficulties. Workers described feeling the heat “in the blood” or that it altered their appearance and voice, making them “drier” or “heavier.”

Most agreed that the heat reduced energy and productivity:

*Well*,* like you sometimes feel uncomfortable because it makes you tired and it makes you lose more energy*,* you know? Because you feel hot and then you lose more energy*,* and you lose… you feel more tired because of the heat. That situation is very bad for anyone. It’s very bad*.

- Male, Almeria 5

In contrast, some workers in Lleida said they had never felt unwell due to heat, though they described occasional headaches as normal.

Participants frequently observed heat-related symptoms in coworkers. Some recounted cases of fainting or vomiting during work, while others mentioned colleagues leaving early because they could not cope with the heat.

*Yes. People faint*,* people vomit from the heat. There was a Moroccan guy… we were working under plastic; it was extremely hot… In the end he couldn’t take it*,* he was vomiting the whole time. Finally*,* we told him to go out*,* because if not he was going to collapse*.

- Male, Almeria 6

Two participants said they knew of workers who had died due to heat, although one death occurred outside the study sites. A few noted hearing coworkers complain frequently about high temperatures, but did not observe others having any symptoms.

Several workers, mainly from Almeria, expressed concern about the long-term health effects of chronic heat exposure:

Yeah, it might affect because the more If you feel it, the more you use more energy, you know? That is a big problem because you need to be healthy in the condition that you survive with the heating.

- Male, Almeria 5

#### Unequal vulnerability to heat-related effects

Participants emphasized that not everyone was affected by heat in the same way. Health status, pre-existing conditions, and age were seen as key determinants of vulnerability.

*Each person is different*,* she may have an illness*,* or may not bear the heat*.

- Female, Huelva 4

Two workers described health conditions that worsened with heat exposure: one had Cutaneous Lupus aggravated by heat and sunlight, another suffered migraines triggered by heat.

Across age groups, participants anticipated that tolerance to heat would decline with age and years of work. One 48-year-old worker said he had never experienced problems but planned to retire by 50 to avoid future risks. Younger workers similarly expected to become more vulnerable as they aged.

Workers also associated long-term agricultural labor with visible and lasting bodily changes. Some recalled coworkers developing hypertension or physical weakness attributed to years in greenhouses.

*Yes*,* I’ve seen it. They don´t*,* they don´t have strength to work. // The skin changes*,* their skin color changes. Even when you leave the greenhouse*,* after four years*,* five years*,* even if you stop working in the greenhouse*,* your skin doesn’t change [back]. It’s always like*,* not black*,* it’s a strange color. It always makes it change*.

- Male, Almeria 12

### Theme 2: The dilemma of heat: balancing work and wellbeing

#### Adverse incentives and economic precarity

Participants generally understood the importance of preventive measures such as taking breaks or resting in the shade. However, economic pressures and workplace dynamics often discouraged them from doing so. Fear of job loss was a central incentive to keep working despite discomfort or symptoms of heat stress. Several workers described being urged to work faster, and in some cases being screamed at when slowing down. In Almeria, some said they would briefly step outside greenhouses to cool off but avoided doing so when supervisors were present, due to the fear of being fired.

*If you want to leave the greenhouse*,* they don’t let you come back to work. There are many who want to work. If you want to work*,* you work*,* if not*,* you don’t come back. And if you need it*,* you manage to work until you get some sickness.*

- Male, Almeria 12

Payment systems also created adverse incentives to continue working despite feeling unwell. Those paid hourly said that leaving early or missing days directly reduced their income. One participant in Huelva recalled a coworker who insisted on continuing to work despite feeling unwell; shortly after, he fainted. For workers contracted abroad, maximizing hours was seen as essential to meet financial obligations.

*For us*,* at least those who are only here for the season*,* it [working more hours] is much better. The more hours we work*,* the better*,* because it means more money. We have to pay housing*,* we have to pay flights. And if we work fewer hours*,* we won’t have any money left to send to Colombia*.

- Female, Lleida 9

Across all study sites, participants agreed that heat made agricultural work difficult but described it as an unavoidable condition that must be endured to sustain a livelihood.

*It’s very hard*,* really it is very hard. But if you*,* if you say I don´t work in the heat. If you don’t have money at home to eat or to pay the house or to send to your wife or your mother too. That´s something else*,* you know?*

- Male, Almeria 11

Some workers framed heat exposure as an inherent aspect of agricultural labor, emphasizing that crops depend on high temperatures. As one worker noted, if plants fail to grow, there is less work available, which would further threaten their income security.

#### Safeguarding wellbeing: Prioritizing health over work

While many workers—especially those without legal documentation—felt constrained in their ability to rest or seek shade, others reported actively choosing to prioritize their health, even when this meant losing income or risking dismissal. Participants described leaving work temporarily or stepping outside to cool off when they felt they might faint.

*If I can’t stand it inside*,* I go outside. Because if you stay there*,* you can collapse.*

- Male, Almeria 9

Several emphasized the importance of staying healthy to be able to continue working in the long term.

*Money is good*,* but I like my body more than money. If I can’t*,* I can’t. I’ll go back home. Because if I work*,* and then I have an illness*,* they’ll only pay me that day. And then I’ll have to spend more buying pills myself*.

- Male, Almeria 10

For some, health-related choices also involved seeking roles that provided greater autonomy. One worker contracted in Colombia said he preferred working in the fields, despite lower pay, because it allowed more flexibility and contributed positively to his mental wellbeing.

### Theme 3: Employer´s efforts to protect workers: A regime of discretion

#### Contingent protection

Participants described substantial variability in heat-related working conditions, largely shaped by employer practices. In Lleida, workers contracted abroad reported receiving annual training at the start of the season, whereas in Almeria and Huelva, formal guidance was typically absent. Adjustments to working hours during summer were common but were usually determined by the employer; only in exceptional cases were they guided by government regulations or allowed worker input.

*So*,* the supervisor sent us home*,* and the next day*,* by government order*,* we could work six hours no more. I did about two weeks*,* only six hours. They called me at six in the morning*,* until one*.

- Male, Lleida 1

Opportunities to take additional breaks or leave early on hot days also varied widely. Some workers could leave when feeling unwell, while others mentioned risking losing their job if doing so. However, in both cases missed hours were usually not paid. Employers occasionally delegated decision-making to workers themselves, but peer pressures could complicate this.

*Sometimes we have to go home earlier*,* but sometimes also*,* if you want to leave*,* others don’t want to leave because if you work*,* you have more money. Also*,* if you don’t work much*,* you don’t get paid much. That’s why you have to stay there*,* work*.

- Male, Almeria 10

This illustrates how the possibility to take heat prevention measures largely depended on employer discretion and how economic precarity could effectively coerce workers into continuing to work despite heat stress. Moreover, short, informal breaks were allowed by some supervisors but not by others. Some participants reported that employers prioritized productivity over worker wellbeing, expecting workers to remain strong and healthy at all times.

*Some people talk to you*,* when you feel a lot of heat*,* go outside. Take 10 min to get a little air*,* then you can go in. But there are bosses who*,* among them are very bad because they don’t want to lose their minute. They are going to say when you can’t [continue working]*,* go home*.

- Male, Almeria 1

The contrast between supervisors who permitted some respite and those who prioritized uninterrupted productivity points to the extent to which workers’ heat protection was contingent on individual managerial disposition rather than any systematic standard.

#### The “Good Boss” / “Bad Boss” dichotomy

Variety in working conditions could to a large extent be attributed to the employer-worker relationship. Many described a distinction between “good” and “bad” bosses, with some stating that the majority did not care about their workers.

*Most of them don’t care about the workers*,* they only care about their products. If the heat affects the crops*,* they do things to reduce the heat so it doesn´t affect the products much. But if the crops need a lot of heat*,* they don’t care about us*,* the ones working here.*

- Male, Almeria 6

Workers with long-term relationships reported greater flexibility in breaks and working hours, whereas short-term or temporary workers were less likely to receive accommodations. Those without a fixed employer more often reported being denied breaks or being scolded. One worker described how he gradually gained more freedom by building trust with an employer:

*The one I worked with for a long time let me [take breaks]. He lets me be well*,* rest and then go back*,* but very few do that. I’ve worked with many different bosses and they’re not all like that. Only two or three*.

- Male, Almeria 12

### Theme 4: Protecting oneself from the heat: Navigating limited agency

Workers described various strategies to cope with heat when employer allowances were limited. These included taking brief, discreet breaks when supervisors were absent or unable to see them, or inventing excuses, such as going to the bathroom or attending to personal matters.

*When … I feel like I can’t take it anymore. For example*,* the boss*,* that someone is not going to let me leave or something*,* I tell him they’re calling me from my son’s school*,* something like that. I make something up. If I know there will be a problem*,* I say something he can’t say no to*.

- Female, Huelva 5

Maintaining hydration was considered essential, though water access was uneven. Most workers brought their own water, and some lacked refrigeration at home, making it difficult to keep water cold. Several participants contracted abroad noted that, according to their contracts, water should have been provided at the workplace, but this was not consistently supplied. For female workers, the absence of toilet facilities—forcing them to urinate in the open fields—sometimes led to reduced fluid intake.

*I don’t drink much water because of that*,* because it makes me pee more. Honestly*,* I hold it in too long before going to the bathroom for that same reason*,* because they could watch us*.

- Female, Lleida 9

Appropriate clothing was another protective strategy. Workers balanced sun protection with comfort, opting for long sleeves, hats, or light materials, and some wetted clothing or head coverings to cool down.

*Yes*,* we wear hats*,* and although the hats*,* some*,* make you*,* make you sweat as well when it’s hot*,* but it’s better to protect yourself from the sun with them and then*,* just like this. This kind of shirt [sleeveless] and shorts*,* yes.*

- Male, Lleida 3

Taken together, the coping practices described across this theme suggest that workers were not passive recipients of inadequate conditions, but active agents drawing on available means to manage their own safety.

### Theme 5: Non-work-related factors: Compounding vulnerabilities

#### Heat at home: precarious housing conditions

Participants reported experiencing heat at home, particularly those living in shanty towns in Almeria and Huelva. Shelters often had low roofs made of plastic, making indoor temperatures unbearable during the day and only cooling at night.

Many workers described difficulty sleeping because of the heat, particularly in the afternoon after work. To escape the heat, some slept outside on balconies or terraces, and one reported sleeping on the floor. A female worker noted that exhaustion sometimes allowed her to sleep despite the heat, but others said it disrupted rest and affected their bodies.

*You get very tired at night if you can’t sleep well*,* you’re always sleepy*,* you know. If you’re sleepy*,* it’s really hot*,* it affects your body to feel like something’s missing*,* you lack sleeping well and that affects your body*,* you know.*

- Male, Lleida 8

In Lleida, some of the workers contracted abroad had air conditioning, but others had to share a single fan or sleep without one. Some participants used fans for coping with heat, while others avoided using them to save on electricity costs.

#### Irregular migrant status: an intersection of risk-factors

Undocumented workers faced amplified risks. Without a work permit, they often struggled to secure stable employment, which limited income and forced many to live in shanty towns. Economic precarity also constrained their ability to take breaks or leave early during hot conditions, as missing work threatened their scarce opportunities.

*To endure it*,* just keep working and that´s it*,* not doing anything. You don’t say*,* “I’m going to rest a bit” or “I’ll go outside.” You just have to endure*,* keep working well*,* and that’s it*,* yes.*

- Male, Lleida 6

This account captures how the absence of legal protections and the precarity of informal employment combined constrained agency even more than for MAW with a formal contract. Workers without contracts often had to work during the summer when formally contracted employees took vacations. One participant described this seasonal disparity:

*Because there are lots of people who*,* when the hot season comes*,* they don’t work*,* they all have vacations. But because you don’t have papers*,* how are you going to have vacations? At that time*,* the people without papers have to keep working… // … And the people who have papers*,* now the boss registers them [in the social security system]. And we are going to work. You are going to go what is most difficult*,* and they come and do what is easy*.

- Male, Almeria 1

This account illustrates how documentation status shaped not only access to rest and time off, but also the allocation of physically demanding and potentially more hazardous work. The intersection of legal invisibility, economic dependency, and occupational exposure meant that undocumented workers were simultaneously the least protected and the most exposed, representing a particularly acute concentration of structural risk factors within an already vulnerable workforce.

## Discussion

This study explored the lived experiences of MAW with occupational heat in Spain. Findings show that heat-related health risks are not merely environmental or occupational issues but are fundamentally shaped by socioeconomic and structural factors. The capacity of most MAW to take preventive actions was limited and often depended on employer discretion, while economic necessity and scarce job opportunities discouraged workers from voicing concerns. Informal and inadequate housing further compounded risks.

Comparable studies in the United States and Europe reveal similar patterns. MAW in Florida, Idaho, and North Carolina described heat as difficult yet unavoidable, emphasizing endurance as part of the job [46–50]. Supervisors’ harassment and pressure to maintain productivity under extreme heat were common, as was fear of job loss when speaking up [46, 48, 51–53]. As in Spain, workers distinguished between “good” and “bad” supervisors, but few received extra breaks during hot days and many felt their safety was disregarded [46–48, 52, 53]. In contrast to Spain, most US workers reported water availability, though some avoided it due to taste, odor, or cleanliness concerns [46, 48, 51, 54, 55]. In California, provision of drinking water and portable toilets by employers is mandatory; implementing these regulations in Spain could improve workers´ hydration [56]. In Cyprus, MAW from low- and middle-income countries exhibited higher body temperatures and fewer unplanned breaks compared to native or higher-income country workers, reflecting greater physical strain and vulnerability [24]. Across settings, economic precarity drives MAW to prioritize income over health, reinforcing patterns of unequal exposure and risk.

Spain’s legal framework aims to protect outdoor workers from excessive heat (Royal Decree 486/1997 and Royal Decree -law 4/2023) [57, 58]. Employers are responsible for risk assessments and mitigation, including adapting working hours or suspending activities during orange or red heat warnings (≥ 39 °C and ≥ 42 °C, respectively) [57]. Local labor conventions vary: in Almeria, farmworkers’ shifts under “abnormal temperatures” are capped at 6 h 20 min daily and 38 h weekly [59], while in Catalonia and Huelva no equivalent measures exist [60, 61]. Labor unions have been calling for heat-prevention and signed agreements with recommendations for prevention with some companies, however, these might not be legally binding [62]. The findings from this study demonstrate that these regulations are insufficient and unevenly applied, leaving workers’ safety largely contingent on employer willingness while enforcement is weak.

Workers in all study sites experienced occupational heat, but greenhouse workers in Almeria and Huelva perceived it as particularly intense. Greenhouse temperatures can exceed outdoor levels, depending on design, ventilation, and crop stage [35, 63, 64]. In greenhouses in Almeria, indoor temperatures can surpass outdoor temperatures by up to 12 °C during midday without ventilation, though proper whitewashing and airflow can reduce this to under 3 °C [63, 65]. Participants also identified whitewashing as an effective mitigation measure and criticized old-design greenhouses lacking ventilation. In Almeria, between April and October, greenhouse workers face some heat stress risk, which increases from June to September with July–August posing “dangerous” conditions [64]. Although activity slows in summer, some work continues during these high-risk periods, underscoring the need for improved environmental protections, including whitewashing and adequate ventilation. Finally, although in Lleida heat was perceived as less of a risk on regular days it remains a serious health risk; notably, two MAW died of heatstroke during heatwaves in the past year [66, 67]. 

The lived experiences of MAW reveal that occupational heat risks cannot be understood solely through environmental exposure. According to the Intergovernmental Panel on Climate Change (IPCC) framework, showing that the risk of suffering adverse health effects from climate change depend on three components: the hazard (heat), the exposure (outdoor / greenhouse work) and individual vulnerability [68]. Vulnerability reflects both individual and structural factors, such as age, health status and socioeconomic position [68]. The concept of structural vulnerability highlights how systemic inequalities, including employment precarity, legal status and gender, shape health risks for MAW [69, 70]. The experiences of MAW in this study exemplify such structural vulnerability: irregular legal status and economic dependency heighten heat-related health risks, while constraining agency to protect oneself. Gender compounds these risks, as shown by female workers who reduced water intake due to lack of toilets or privacy, and prior reports of gender-based abuse in Huelva [37, 71]. These findings underscore how social marginalization amplifies MAW´s vulnerability to heat.

Another factor that could impact structural vulnerability is ethnicity. In Spain, and Europe in general, studies largely operationalize inequality using migration status and data on race or ethnicity is rarely collected [72, 73], even though Spain is home to one of Europe’s largest Roma populations [74]. Other ethnic minorities mainly consist of recent immigrants and their descendants [74]. Due to the scarcity of ethnic and racial data and the close intertwining of ethnicity and migration background in the Spanish context, it is difficult to assess the independent impact of ethnicity on labor conditions and health outcomes. The absence of such data has been criticized by activists and researchers as it also prevents adequate monitoring of ethnic discrimination [74]. From 2026 onwards, Spain plans to include equality data based on racial or ethnic origin in national surveys [75]. Therefore, future studies should take into account the role of ethnicity on heat-related health outcomes.

In addition to labor regulations, migration laws also shape structural vulnerability of MAW. An irregular legal status often prevents migrants from improving their working conditions [76]. The Spanish government has recently taken steps to facilitate regularization. In May 2025, a reformed immigration law (Royal Decree 1155/2024) entered into force, allowing migrants to apply for residency after two years of residence instead of three [77]. In addition, an exceptional regularization for migrants who arrived before the end of 2025 and can prove at least five months of residence is expected to benefit approximately 500,000 individuals [78]. These measures may reduce vulnerability by improving access to legal employment, although even after reforms, regularization still requires long periods of informal residence. In addition, difficulties obtaining proof of residence or employment has been widely reported and limits access to regularization for the most vulnerable workers [79]. The reformed law also allows holders of the “social-labor integration permit” (arraigo sociolaboral) to change employers after three months without justification, reducing dependence on a single employer [77]. However, workers contracted through the Collective Management of Recruitment at Source (*gestión colectiva de contrataciones en origen*; GECCO) system remain tied to one employer and can only change jobs under exceptional circumstances [42]. Despite recent improvements, further reforms are needed to ensure that immigration policies effectively reduce MAW’s structural vulnerability.

### Strengths and limitations

This study had some limitations. First, linguistic limitations excluded some potential participants, as most interviews were conducted in English or Spanish. Two interviews were conducted in Darisha with translation to include Moroccan women, but expanding to additional languages was not feasible. In addition, as most interviews were not conducted in participants´ native languages, this might have limited their ability to express themselves fully. Second, recruitment through local civil society organizations may also have influenced responses due to social desirability bias, despite clear assurances of confidentiality and voluntary participation, and despite NGO staff not being present during interviews. However, this approach also increased trust between the participants and the interviewer which might have led to more openness. A strength of this study is the direct engagement with MAW voices, contrasting prior research that often centers professionals’ perspectives [79–81]. The inclusion of diverse participants across three sites and multiple nationalities allowed for rich comparative insights.

### Implications and recommendations

Although Spanish regulations legally require employers to assess and mitigate occupational heat risks, the type and extent of preventive measures are largely left to employer discretion. This study shows that such flexibility often results in inconsistent protection, particularly for the most precarious workers. Regulations should therefore specify clearer, enforceable standards, such as defined maximum allowable temperatures for agricultural work and mandatory minimum rest breaks. Enforcement mechanisms must also be strengthened through increased labor inspections. In addition, labor inspections should be decoupled from immigration enforcement, so that undocumented workers can report unsafe conditions without facing the risk of deportation. Beyond regulatory reform, interventions should address the structural determinants of heat vulnerability. Improving housing conditions, expanding access to water and sanitation, and ensuring MAW´s legal protections are essential. This includes implementing policies that ensure affordable (temporary) housing, rather than abolishing the settlements where MAW live, without providing alternative housing options. Addressing these broader inequities aligns with both occupational safety and climate justice frameworks, recognizing that MAW’s vulnerability to heat is embedded in intersecting social, legal, and economic disadvantages.

## Conclusion

This study suggests that MAW in Spain face occupational heat stress shaped by precarious work, limited housing, and unequal power relations. Their lived experiences reveal that MAW navigate a dilemma between protecting their health and sustaining their livelihoods, while their protection depends on the arbitrary goodwill of employers rather than enforceable rights. To reduce these risks, Spain’s occupational heat regulations must include clearer preventive measures, stronger enforcement, and broader structural interventions that improve working and living conditions. Without such changes, MAW will remain disproportionately exposed to the growing dangers of heat stress in a warming climate.

## Annex 1: Interview guide

Live path and living and working conditions.

1. Could you tell me about how you got to Spain? (i.e., when, how, why)
2. Could you tell me about your current situation living in Spain?
  - Living at one place or moving around
  - Type of housing and facilities (e.g. apartment, informal housing, housing provided by employer, electricity, running water, air-conditioning etc.)
  - Explore working conditions
    i. What type of contract do you have? (e.g. compliance with contract, secured number of hours or much insecurity, risk of losing job etc.)
    ii. What does your working day look like? (e.g. breaks, tasks you perform, how is your relationship with your supervisor)

Health and healthcare

3. How do you consider your health? How is your body?
4. What do you do to take care of your health and your body?
5. What do you do to take care of your mind?
6. How is your work affecting your health? (body and mind)
7. How do you sleep?
8. Do you worry a lot? What are you worried about?
9. In your country of origin, where did you use to go for healthcare? (healthcare center or traditional healthcare)
  - What kind of services were provided to you? (e.g. only acute treatment or also preventive? Only pills / medication or also tests?)
10. In Spain, where do you go for healthcare?
  - What is your experience? Do you feel you are being helped well? (e.g. are your health issues being solved, are you being treated well?)
  - Did you phase any problems when trying to access healthcare?

Occupational heat stress

11. How do you consider the weather in this country? How does it affect you? (open question not yet steering towards heat)
12. How does the weather affect you when working in summer? (e.g. does the heat make you uncomfortable or are you used to it, do you see it as a problem, are you worried about it)
13. Could you describe a working day when it is very hot? (e.g. how does it make you feel, what are the working hours, when do you take breaks, drink water etc.)
  - What does your employer do to reduce the impact of heat? (e.g. is the schedule different then normal, do you get extra breaks, do they give you instructions etc.)
  - What do you do to reduce the impact of heat? (e.g. change clothing, drink more, work slower etc.)

14. Do you think the heat when working in summer impacts your health and wellbeing? If so, can you tell me in what way? (e.g. do you feel more tired, less productive)
  - Did you ever feel ill while working in the heat? if so, can you describe this experience?
  - Did you ever see co-workers becoming ill when working in the heat? Can you describe what you saw or what they told you?

15. Did you ever feel bad and wanted to see a doctor after working in the heat?
  - If so, what did you do? (e.g. did you or ask someone for help or was their a reason not to)
  - If you went to see a doctor, how was the experience? How was the process of reaching the doctor (e.g. how did you go to the healthcare center / place you received care, did anyone come with you or translate for you if needed, did you have any problems, such as not having the right documents or not understanding the process etc.) Were you helped well?

16. Is there anything else you want to say about the impact of heat during work? Do you have any suggestions?
17. Is there anything else that´s important for me to know to better understand your situation?

## Annex 2: Original quotes in Spanish

**Table:** 

Original quote	Translation
Bueno, yo lo aguanto porque soy africano. Tenemos ya el calor en África todo el año. Es verano todo el año. Entonces a mí no me sorprende el calor, no me sorprende.	Well, I can stand it because I’m African. We already have heat in Africa all year round. It’s summer all year. So the heat doesn’t surprise me, it doesn’t surprise me.
Porque el plástico hace mucho calor. Si afuera hay 30, adentro, casi 40.	Because the plastic makes it very hot. If it’s 30 degrees outside, inside it’s almost 40.
Te sientes fatal. Te esperas la hora que te termina y viene corriendo a tu casa a duchar y descansar. Eso. Todo el día echamos el agua encima de la cabeza, entrar y así. Todo sufre con la temperatura, pero ¿qué hacemos? Tenemos que trabajar.	You feel terrible. You wait for your shift to end and you come running home to shower and rest. That’s it. All day we pour water over our heads, go in and out like that. Everything suffers from the heat, but what can we do? We have to work.
El año pasado esto fue fuerte, fuerte bastante. Mucho golpe de calor, mucho calor. Y bueno, entonces ya por la noticia salía que, hasta muertes, que había uno que por la noticia. Y un día estábamos trabando, ese día, mejor dicho, tomé agua y buscando la sombra porque no daba más.	Last year it was bad, pretty bad. A lot of heat stroke, a lot of heat. And well, it came out in the news that, even deaths, there was one in the news. And one day we were working, that day, I mean, I had to drink water and look for shade because I couldn’t take it anymore.
Bueno, tiempo de calor de veces tú puedes trabajar en campo, hace mucho calor, tu cabeza te duele, bueno, llega en casa también contra calor, entonces la cabeza puede seguir duele, entonces tú puedes tomar pastillas para que tú puedes dormir un poco.	Well, during hot times you can sometimes work in the field, it’s very hot, your head hurts, well, arriving home you also find heat, so your head can keep hurting, and then you can take pills so you can sleep a little.
Dentro del invernadero es como un shock, como que trabajando sin… A veces trabajando no sabes qué haces, solo trabaja, porque es mucho calor.	Inside the greenhouse it’s like a shock, like working without… Sometimes when working you don’t know what you’re doing, you just work, because it’s so hot.
Sí. A la gente que desmayan, a la gente que vomitan por el calor. Un tío maroqui, estamos trabajando con plástico, estaba muchísimo calor. Al final no aguantaró, estaba todo el rato vomitando. Al final, le dijimos que sale, porque si no se va a caer.	Yes. People faint, people vomit from the heat. There was a Moroccan guy… we were working under plastic; it was extremely hot… In the end he couldn’t take it, he was vomiting the whole time. Finally, we told him to go out, because if not he was going to collapse.
Sí, he visto. No tienen, no tiene fuerza de trabajar. Si los pelos, quitar pelo también afecta al pelo, también, afecta el pelo, el calor. Y no tienen fuerza de trabajo más. Y piel, cambia piel. Con muchos años cambia piel también. Ser más… No sé cómo. Cambia piel, su color de piel cambia. Aunque sales del invernadero, cuando estás aquí cuatro años, cinco años, Aunque para en invernadero no cambia la piel. Siempre es como no es negro, es un color raro. Y hace siempre la cambia.	Yes, I’ve seen it. They don´t, they don´t have strength to work. Yes, the hairs, taking away the hairs, it affects the hairs too, the heat. And they don’t have the strength to work more. And the skin, it changes the skin. After many years the skin changes too. Being more… I don’t know how. The skin changes, their skin color changes. Even when you leave the greenhouse, after four years, five years, even if you stop working in the greenhouse, your skin doesn’t change [back]. It’s always like, not black, it’s a strange color. It always makes it change.
Si quieres salir de invernadero, te dejan que no volvieses a trabajar. Hay muchos que quieren trabajar. si quieres trabajar, trabajar, si no, no vuelves. Y si necesitas, consigues trabajar hasta que tienes alguna enfermedad.	If you want to leave the greenhouse, they don’t let you come back to work. There are many who want to work. If you want to work, you work, if not, you don’t come back. And if you need it, you manage to work until you get some sickness.
Para nosotros, por lo menos que estamos solo la temporada, para nosotros es muchísimo mejor. Entre más horas trabajemos, mejor, porque más dinero, nosotros tenemos que pagar alojamiento, tenemos que pagar vuelos. Y si hacemos menos horas, pues no nos va a quedar dinero para mandar para Colombia.	For us, at least those who are only here for the season, for us it’s much better. The more hours we work, the better, because it means more money. We have to pay housing, we have to pay flights. And if we work fewer hours, we won’t have any money left to send to Colombia.
Es muy difícil, la verdad es muy difícil. Pero si tú también, tú dices que yo no trabajo con el calor. Si tú no tienes dinero en casa para comer o para pagar la casa o para mandar a tu mujer o tu madre también. Es otra cosa, ¿sabes?	It’s very hard, really it is very hard. But if you, if you say I don´t work in the heat. If you don’t have money at home to eat or to pay the house or to send to your wife or your mother too. That´s something else, you know?
Si yo no puedo aguantar dentro, salgo fuera. Porque si te quedas ahí, se puede caer.	If I can’t stand it inside, I go outside. Because if you stay there, you can collapse.
Dinero es bien, pero me gusta mi cuerpo más que dinero. Si no puedo, no puedo. Voy a volver a casa. Porque si trabajo, ahí si tengo enfermedad, ellos van a pagarme solo este día. Y luego yo voy a cobrar más para comprar pastillas solo yo.	Money is good, but I like my body more than money. If I can’t, I can’t. I’ll go back home. Because if I work, and then I have an illness, they’ll only pay me that day. And then I’ll have to spend more buying pills myself.
Entonces, ya el encargado nos mandó a casa y ya para el día siguiente y ya por orden del Gobierno, matar seis horas nada más. Yo hago como dos semanas, apenas seis horas. Me llamo a las seis de la mañana, a la una.	So, the supervisor sent us home, and the next day, by government order, we could work six hours no more. I did about two weeks, only six hours. They called me at six in the morning, until one.
A veces tenemos que volver a casa más pronto, pero a veces también, si tú quieres salir, otras personas no quieren salir porque como tú trabajas, como tienes más dinero. También, si no trabajas mucho, no vas a cobrar mucho. Por eso tienes que estar ahí, trabajar.	Sometimes we have to go home earlier, but sometimes also, if you want to leave, others don’t want to leave because if you work, you have more money. Also, if you don’t work much, you don’t get paid much. That’s why you have to stay there, work.
Hay gente que vas a hablar contigo, cuando tú te sientes calor mucho, sale fuera. Hace 10 minutos para tomar aire un poco, después tú puedes entrar. Pero hay jefes que, dentro de ellos es muy mal porque ellos no quieren perder su minuto. Vas a hablar cuando tú no puedes [seguir trabajando], marchar a casa.	Some people talk to you, when you feel a lot of heat, go outside. Take 10 min to get a little air, then you can go in. But there are bosses who, among them are very bad because they don’t want to lose their minute. They are going to say when you can’t [continue working], go home.”
La mayoría de ellos no le importa los trabajadores, solo le importa sus productos. Si el calor afecta los productos, hace cosas para disminuir el calor que no afecta mucho los productos. Pero si los productos van a necesitar mucho calor, no les importa a nosotros, a la que trabaja en aquí.	Most of them don’t care about the workers, they only care about their products. If the heat affects the crops, they do things to reduce the heat so it doesn´t affect the products much. But if the crops need a lot of heat, they don’t care about us, the ones working here.
Lo que trabajo mucho tiempo con él, me dejó [tomar descansos]. Me dejó y estar bien, descansado y volver, pero es muy pocos los que hacen eso. Yo trabajo con muchos jefes diferentes y no es todo así. Es dos o tres.	The one I worked with for a long time let me [take breaks]. He lets me be well, rest and then go back, but very few do that. I’ve worked with many different bosses and they’re not all like that. Only two or three.
Cuando, sí, por ejemplo, cuando siento que estoy y no puedo, eso. Por ejemplo, el jefe, que una persona que no va a dejarme salir o eso, yo digo a él que me llama al colegio de mi hijo, eso, eso. Algo de mentira es algo. Si se sabe que va a tener un problema, lo digo las cosa que sé que no me puede decir que no.	When … I feel like I can’t take it anymore. For example, the boss, that someone is not going to let me leave or something, I tell him they’re calling me from my son’s school, something like that. I make something up. If I know there will be a problem, I say something he can’t say no to.
Voy poco a tomar agua por eso, porque me da más chichi. La verdad, me aguanto demasiado para ir al baño por lo mismo, porque de pronto nos miran.	I don’t drink much water because of that, because it makes me pee more. Honestly, I hold it in too long before going to the bathroom for that same reason, because they could watch us.
Si, llevamos sombreros y aunque los sombreros algunos te dan calor, te dan sudor también cuando hace calor, pero es mejor protegerse del sol con esto y luego solo con esto. Este tipo de camisa [sin mangas] un pantalón corto, pues sí.	Yes, we wear hats, and although the hats, some, make you, make you sweat as well when it’s hot, but it’s better to protect yourself from the sun with them and then, just like this. This kind of shirt [sleeveless] and shorts, yes.
Tú cansas muchos por la noche si no puedes dormir bien siempre tú tienes sueño sabes. Si tú tienes sueño, hace muchísimo calor va a afectar que tú vas a sentir a tu cuerpo que falta algo, te falta dormir bien por eso que se afecta en cuerpo sabes.	You get very tired at night if you can’t sleep well, you’re always sleepy, you know. If you’re sleepy, it’s really hot, it affects your body to feel like something’s missing, you lack sleeping well and that affects your body, you know.
Para aguantar solo de trabajar y ya está si no hacer nada. Tú no dices me va a descansar un poco, tú no vas a hacer me va a afuera. Quiere que aguantar, quiere aguantar solo trabajar bien y ya está sí.	To endure it, just keep working and that´s it, not doing anything. You don’t say, “I’m going to rest a bit” or “I’ll go outside.” You just have to endure, keep working well, and that’s it, yes.
Porque hay un montón de gente que cuando llega el tiempo del calor, ellos no trabajan, todo tiene vacaciones. Como tú no tienes papeles, ¿cómo vas a tener vacaciones? Esos tiempos la gente que no tienen papeles tienen que seguir trabajando… // … Y la gente que tiene papeles. Ahora, el jefe va a dar de alta para ellos. Y nosotros vamos a trabajar. Tú vas a hacer lo que está muy difícil, y ellos vienen a hacer lo que está fácil.	Because there are lots of people who, when the hot season comes, they don’t work, they all have vacations. But because you don’t have papers, how are you going to have vacations? At that time, the people without papers have to keep working… // … And the people who have papers, now the boss registers them [in the social security system]. And we are going to work. You are going to go what is most difficult, and they come and do what is easy.

Quotes that are not included in this table were originally in English

## Data Availability

De-identified data that support the findings of this study are available from the corresponding author upon reasonable request and with permission from the authors’ institution.

## Abbreviations

EU: European Union
GDPR: General Data Protection Regulation
HRI: Heat-Related Illness
IPCC: Intergovernmental Panel on Climate Change
MAW: Migrant Agricultural Worker(s)
NGO: Non-Governmental Organization
WBGT: Wet Bulb Globe Temperature
